# Artificial intelligence-based personalized nutrition and prediction of irritable bowel syndrome patients

**DOI:** 10.1177/17562848221145612

**Published:** 2022-12-26

**Authors:** Animesh Acharjee, Saptamita Paul Choudhury

**Affiliations:** Institute of Cancer and Genomic Sciences and Centre for Computational Biology, College of Medical and Dental Sciences, University of Birmingham, Birmingham B15 2TT, UK; Institute of Translational Medicine, University Hospitals Birmingham NHS Foundation Trust, Birmingham, UK; NIHR Surgical Reconstruction and Microbiology Research Centre, University Hospitals Birmingham, Birmingham, UK; MRC Health Data Research UK (HDR UK), London, UK; Department of Human Genetics, National Institute of Mental Health and Neurosciences, Bengaluru, India

Chronic functional gastrointestinal disorder irritable bowel syndrome (IBS)^1^ has a detrimental effect on people’s quality of life and access to healthcare. IBS is considered one of the most common intestinal discomforts and pains that pose a substantial risk to public health. IBS has complicated pathogenesis; however, the current research indicates that the gut microbiota may be crucial for the initiation, continuation and intensity of such problems.^2^ According to one popular notion, an abnormality within gut microbiome causes stimulation of the intestinal immune response and possibly low-grade swelling.^3–5^ Another important information confirming this theory is an elevated probability of acquiring IBS following perturbations of the gut microbiome. Nevertheless, because of different and varying microbial patterns across individuals, identifying diagnostic biomarkers^6^ for IBS may be difficult. The other explanation for this disparity may be because the microbiome’s variations hinder analysis process during intestinal bacterial research across time. As a result, a glimpse of cross-sectional study findings loses chronological precision and does not depict clinical aspects of IBS.^7^

With the recent development of next-generation sequencing, it has been shown that changes in the gut microbiome are linked to IBS. Observational studies have consistently demonstrated that the makeup of the gut microbiota changes in the context of IBS. Microbiomes such as *Proteobacteria* and *Streptococcus* levels in faeces and the gut mucosa were shown to be higher in abundance in several studies. Diet is becoming a more and more popular interventional strategy for treating IBS as it significantly affects the gut microbiome abundance. One effective dietary intervention for IBS is the low fermentable oligosaccharides, disaccharides, monosaccharides and polyols diet.^8^

A very recent article by Karakan *et al*. (2022)^9^ reported an interesting study where they considered a total *n* = 25 baseline group of IBS patients, and the healthy controls (*n* = 34) were compared in terms of their microbiota compositions. Out of *n* = 25 patients, 6 weeks of a personalized nutrition diet (*n* = 14) for group 1 and a standard IBS diet (*n* = 11) for group 2 were followed and then compared. A schematic diagram is presented in Figure 1. The individualized nutrition model was developed by the artificial intelligence model called XGBoost (Extreme Gradient Boosting) and IBS index scores were produced. XGBoost is a technique for group learning. It might not always be enough to rely solely on a single machine learning model’s output. A methodical approach to combining the prediction capacity of various learners is provided by ensemble learning. A single model that provides the combined output from multiple models is the final outcome. Due to ensemble technique, it improved speed and performance. The score distributions of IBS patients and healthy controls differ significantly (*p* = 0.001), which suggests that the machine-learned IBS index is an important predictor of the disease. Personalized nutrition showed a statistically significant rise in the *Faecalibacterium* genus (*p* = 0.04), whereas an increasing trend in *Prevotella* (*p* = 0.057) was noted in the standard IBS diet group. In this analysis, researchers also found an elevation in *Bacteroides* within personalized nutrition cohort (*p* > 0.05). The elevation with in *Bacteroides* grouping may have influenced the IBS individuals’ stress levels in the intervention group, improving their performance levels in the Irritable Bowel Syndrome Severity Scoring System test. Meydan *et al.* demonstrated that highly precise dietary therapies using prebiotics and probiotics directed by metagenomic research succeeded in clinical alleviation as well as related microbiome compositional alteration.^10^

**Figure 1.**
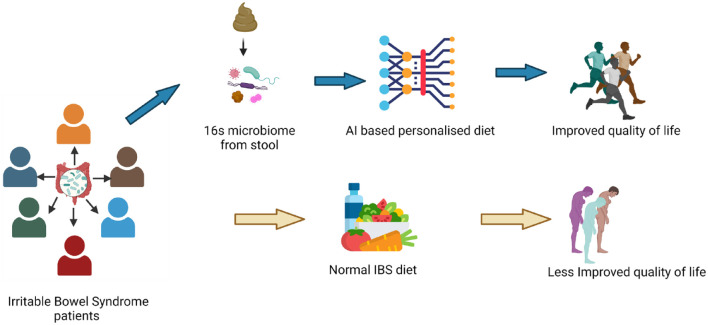
The overall workflow of the entire process is represented as a schematic diagram.

There is currently no ideal or limited diet for treating IBS patients as IBS is a heterogeneous set of diseases and hence might reflect the changes in gut microbiome. The optimum diet likely to be specific for each individual patients. According to majority estimations, diets are a stronger predictor of individual differences in the makeup of the intestinal microbiome than genetics. This study could be the initial effort to achieve these treatment objectives in IBS patients based on diet intervention. This research also highlights the diagnostics^6^ and therapeutic impact of a customized diet on each person’s gut flora and disease-specific symptoms promoting personalized and translational research in IBS.

## Declarations

**Ethics approval and consent to participate:** Not applicable.

**Consent for publication:** Not applicable.

**Availability of data and materials:** Not applicable.
